# Increased expression of cystine/glutamate antiporter in multiple sclerosis

**DOI:** 10.1186/1742-2094-8-63

**Published:** 2011-06-03

**Authors:** Olatz Pampliega, María Domercq, Federico N Soria, Pablo Villoslada, Alfredo Rodríguez-Antigüedad, Carlos Matute

**Affiliations:** 1 Neurotek-UPV/EHU, Parque Tecnológico de Bizkaia, Zamudio, Bizkaia, Spain; 2 Centro de Investigación Biomédica en Red en Enfermedades Neurodegenerativas (CIBERNED), Departamento de Neurociencias, Universidad del País Vasco, Leioa, Bizkaia, Spain; 3 Departamento de Neuroinmunología, Hospital Clínic - IDIBAPS, Barcelona, Spain; 4 Servicio de Neurología, Hospital de Basurto, Bilbao, Bizkaia, Spain; 5 Department of Developmental and Molecular Biology, Albert Einstein College of Medicine, Bronx, New York, NY, USA

## Abstract

**Background:**

Glutamate excitotoxicity contributes to oligodendrocyte and tissue damage in multiple sclerosis (MS). Intriguingly, glutamate level in plasma and cerebrospinal fluid of MS patients is elevated, a feature which may be related to the pathophysiology of this disease. In addition to glutamate transporters, levels of extracellular glutamate are controlled by cystine/glutamate antiporter x_c_^-^, an exchanger that provides intracellular cystine for production of glutathione, the major cellular antioxidant. The objective of this study was to analyze the role of the system x_c_^- ^in glutamate homeostasis alterations in MS pathology.

**Methods:**

Primary cultures of human monocytes and the cell line U-937 were used to investigate the mechanism of glutamate release. Expression of cystine glutamate exchanger (xCT) was quantified by quantitative PCR, Western blot, flow cytometry and immunohistochemistry in monocytes in vitro, in animals with experimental autoimmune encephalomyelitis (EAE), the animal model of MS, and in samples of MS patients.

**Results and discussion:**

We show here that human activated monocytes release glutamate through cystine/glutamate antiporter x_c_^- ^and that the expression of the catalytic subunit xCT is upregulated as a consequence of monocyte activation. In addition, xCT expression is also increased in EAE and in the disease proper. In the later, high expression of xCT occurs both in the central nervous system (CNS) and in peripheral blood cells. In particular, cells from monocyte-macrophage-microglia lineage have higher xCT expression in MS and in EAE, indicating that immune activation upregulates xCT levels, which may result in higher glutamate release and contribution to excitotoxic damage to oligodendrocytes.

**Conclusions:**

Together, these results reveal that increased expression of the cystine/glutamate antiporter system x_c_^- ^in MS provides a link between inflammation and excitotoxicity in demyelinating diseases.

## Background

Multiple sclerosis (MS) is a chronic, degenerative disease of the CNS, which is characterized by focal lesions with inflammation, demyelination, infiltration of immune cells, oligodendroglial death and axonal degeneration [1-3]. MS is typically considered as a primary inflammatory disease in the early, relapsing phase which progresses to a secondary, progressive stage that is characterized by a diminished inflammatory activity and global brain atrophy [4].

Oligodendroglial death and demyelination can occur through glutamate excitotoxicity [5,6], a phenomenon that takes place when an excessive amount of glutamate overactivates ionotropic glutamate receptors (iGluRs). Several observations have linked glutamate excitotoxicity with MS demyelination. First, experimental autoimmune encephalitis (EAE), an animal model for MS, is ameliorated by AMPA and kainate iGluR antagonists, improving oligodendrocyte loss and demyelination without affecting immune reaction [7-9]. And second, the infusion of glutamatergic agonists into rabbit optic nerve leads to inflammation, oligodendrocyte loss, demyelination, and axonal damage, reminding these characteristics those typical lesions in MS [10].

Data supporting the excitotoxic hypothesis in MS include the report of higher glutamate levels in MS, both at CNS and peripheral blood. Glutamate is increased in cerebrospinal fluid (CSF) from MS patients with acute lesions, whereas in silent ones glutamate is similar to controls [11]. Glutamate is also increased in acute MS lesions and in normal-appearing white matter in MS patients [12]. Finally, glutamate plasma levels are also increased in relapsing MS patients [13]. Together, these data point to an implication of glutamate excitotoxicity in MS pathology [14,15].

Extracellular glutamate increase in the CNS may originate from brain blood barrier breakage during pathological conditions [16]. In blood, monocytes are able to release glutamate, but not lymphocytes [13]. Monocytes participate in the regulation of intrathecal inflammation observed in MS, and constitute the major cell type in the perivascular infiltrates that are characteristic of MS. In addition, CD11b^+^CD115^+^ly-6C^high ^monocytes are precursors of CNS dendritic cells and macrophages in EAE lesions, being dynamically regulated during the course of EAE and accumulating in blood immediately prior to clinical relapses [17]. Thus, monocytes contribute to the pathological-anatomical features in the CNS of MS patients [18] and are an attractive tool for understanding some of the CNS alterations that occur in MS. In addition, their easily follow-up from blood constitutes a useful characteristic to use these cells as biomarkers for MS.

We have therefore analyzed the role of monocytes in the alteration of glutamate homeostasis in MS pathology. One of the regulators of extracellular glutamate is the cystine/glutamate antiporter [19], also termed system x_c_^-^, a heterodimer composed of two subunits xCT and 4F2hc. The xCT light chain confers the specificity of amino acid transport, whereas the ubiquitously expressed 4F2hc is common to other amino acid transport and is required for membrane expression of xCT. The cystine/glutamate antiporter is a chloride-dependent, sodium-independent transporter, whose main function is to provide cystine for antioxidant glutathione synthesis [20]. We demonstrated that activation of human monocytes induces glutamate release through system x_c_^- ^and an increase in the expression of its catalytic subunit xCT. Moreover, we provide evidence that xCT expression is increased in monocyte-macrophages-microglia lineage in EAE and MS, both at CNS and peripheral blood, suggesting a link between glutamate excitotoxicity and inflammation in MS.

## Methods

### Human samples

Peripheral blood for RNA expression studies was obtained from the Neurology Service of the Hospital of Basurto. Healthy controls matched by sex and age were recruited at the University of the Basque Country. Characteristics of peripheral blood samples are described in Table 1. All the MS patients used in this study suffered the relapsing form of the disease (R-MS), which included relapsing-remitting (RR) and secondary-progressive (SP) subtypes. Thirty five per cent of all the patients were under long term treatment with immune-suppressors. Among R-MS patients, 24 were experiencing a clinical relapse and were not being treated with steroids at the time of blood sampling. All subjects gave informed consent to participate in the study. The study was approved by the local ethical committee.

**Table 1:** Characteristics of human blood samples.

Leukocytes			
	***Gender F/M (n)***	***Age (Mean ± SD)***	***RR-SP***

*MS in remission*	25/3	36.07 ± 8.48	(20-8)
		Range	
		(20 - 51)	
*MS in relapse*	19/5	33.15 ± 7.57	(21-3)
		Range	
		(19 - 50)	
*Controls*	20/5	33.64 ± 8.69	
		Range	
		(19 - 51)	

F, female; M, male; RR, relapsing-remitting MS; SP, secondary-progressive MS.

For RNA expression studies, postmortem optic nerve samples from 16 long-standing MS patients and 12 control subjects (who died from non-neurological diseases) were obtained at autopsy under the management of the Netherlands Brain Bank. These samples have been previously characterized ([21]; see Table 2). MS samples were classified according to clinical data together with macroscopic tissue analysis as normal appearing (NAON) or damaged optic nerves (DON).

**Table 2:** Clinical characteristics of human optic nerve samples.

Reference	Code	Gender	Age	PMD (hours)	Duration of MS (years)	MS type	Optic Nerve Alterations
*MS Samples*							
MS1	98-176	M	83	7:05	52	PP	No
MS2	99-054	F	58	8:10	20	SP	Yes
MS3	98-185	F	70	8:55	19	PP	Yes
MS4	98-158	F	76	16:31	53	SP	Yes
MS5	98-087	F	55	7:35	> 11	SP	No
MS6	98-179	F	60	8:50	36	PP	Yes
MS7	99-062	F	79	10:00	44	PP	No
MS8	99-025	F	64	7:45	35	SP	Yes
MS9	98-066	F	75	4:50	> 27	PP/SP	No
MS10	01-018	F	48	8:10	8	SP	No
MS11	01-003	M	69	10:00	34	PP	Yes
MS12	99-119	F	38	5:15	10	RR	No
MS13	00-024	F	52	8:25	16	PP/SP	Yes
MS14*	99-109	M	70	6:25	22	SP	Yes
MS15*	99-121	M	51	7:50	29	SP	Yes
MS16*	99-066	M	69	16:45	46	PP	No
*Control Samples*							
C1, 14,16	98-101	M	72	6:45			
C2,5	98-125	F	58	6.15			
C3	98-104	F	74	7:25			
C4	98-051	F	94	16:50			
C6	98-061	F	64	6:00			
C7	98-042	F	79	9:45			
C8	98-148	F	54	5:35			
C9	98-112	F	84	9:20			
C10, 13	00-050	F	52	6:50			
C11	00-090	M	70	7:45			
C12	00-025	F	41	13:30			
C15	98-127	M	56	5:25			

Optic nerve alterations consisted of macroscopic plaques, atrophy and/or optic neuritis.

Abbreviations: Reference, Netherlands Brain Bank bar-coded numbers; A, microarrays; qPCR, real-time quantitative PCR; IH, immunohistochemistry; C, control; MS, multiple sclerosis; M, male; F, female; PMD, postmortem delay; PP, primary progressive; SP, secondary progressive; RR, relapsing remitting. * These samples have been used only for immunohistrochemistry

For immunohistochemistry studies, additional postmortem spinal cord samples from MS and control subjects were obtained at autopsy from the Netherlands Brain Bank. Characteristics of spinal cord samples are described in Table 3. Frozen tissue was kept at -80°C until use.

**Table 3:** Characteristics of human spinal cord samples.

Spinal Cord					
	***NBB reference***	***Gender***	***Age***	***Neuropathology***	***Postmortem time (h)***

*MS*	99-109	Male	70	MS with no active plaques	6:25
	99-119	Female	38	MS with active plaques	5:15
	99-121	Male	51	MS	7:50
	00-024	Female	52	MS	8:25
					
*Controls*	00-025	Female	41		13:30
	00-050	Female	52		6:50
	00-090	Male	70		7:45
	02-008	Male	62		9:35

For comparisons, all MS samples were matched with control samples for sex, age and post-mortem delay.

### Human PBMC, monocyte and U-937 cell line culture

Blood was taken into heparin-coated tubes and peripheral blood mononuclear cells (PBMCs) were isolated by centrifugation over Ficoll-Paque PLUS (GE Healthcare). For flow cytometry analysis PBMCs were plated at 1.5 × 10^6 ^cells per ml in complete RPMI media with GlutaMax and 25 mM HEPES (Gibco), and supplemented with 4.5 g/l glucose, 1 mM sodium pyruvate, 10% heat-inactivated FBS and 1% penicillin-streptomycin. Monocytes were purified from isolated PBMCs by MACS using the Monocyte Isolation Kit II (Milteny Biotec) following manufacturer's instructions. Cells were plated at 0.5 × 10^6 ^cells per ml in complete RPMI media for 72 h. Then, the day before glutamate release experiments media was changed to DMEM with 1% of heat-inactivated FBS and 1% of penicillin-streptomycin. Human U-937 monocytic cell line (ATCC) was cultured in suspension in complete RPMI media supplemented as before. Drugs were added 30 min before cell activation by LPS (1 μg/ml; *Escherichia coli *O11:B4; Sigma-Aldrich), and 48 h after, cells and culture supernatants were collected for experiments.

### Induction and treatment of acute and chronic EAE

Rats (Male Lewis rats, 8 week-old weighing 200-220 g) were injected subcutaneously in both hind feet with inoculum containing 100 μg of guinea pig myelin basic protein (Sigma) diluted in water, emulsified in equal volumes of Freund's incomplete adjuvant (Sigma), supplemented with 500 μg of heat-inactivated *M. tuberculosis *H37Ra (DIFCO Laboratories). The neurological deficits started 10 days postimmunization (dpi), peaked after 14 days and remitted by 20 dpi. Neurological impairment was monitored and scored daily according to the following scale ranging from 0 to 8: 0, normal; 1, flaccid tail; 2, tail paralysis; 3, loss of muscle tone in hindlimbs; 4, hindlimbs hemiparalysis; 5, complete hindlimbs paralysis; 6, moderate paraparesis; 7, tetraparalysis; 8, death. The tissues and samples examined in this study were obtained at 14 dpi from rats showing a neurological score between 3 and 6.

Chronic, relapsing EAE was induced in C57BL/6 mice by immunization with 300 μl of MOG(35-55) (200 μg; Sigma, Barcelona, Spain) in incomplete Freund's adjuvant supplemented with 8 mg/ml *Mycobacterium tuberculosis *H37Ra. Pertussis toxin (500 ng; Sigma) was injected on the day of immunization and again two days later. Neurological impairment was monitored as in acute EAE. Mice were sacrificed at 20 days after immunization, when maximal motor symptoms were detected (see Additional file 1, Figure S1).

### Measurement of glutamate release and glutathione

Glutamate release from U-937 and human primary monocytes was monitored by an enzymatic assay based on the activity of the enzyme L-glutamate dehydrogenase (15 U/ml; Sigma-Aldrich) as previously described [13,22]. Glutamate was oxidized by L-glutamate dehydrogenase in the presence of 1 mM NADP^+ ^to α-ketoglutarate with the formation of NADPH and fluorescence emission at 430 nm (excitation light 335 nm). Release was quantified in reference to standard curves constructed with exogenous glutamate. In each assay, 20 μl of culture supernatant were used in a final volume of 300 μl.

Intracellular reduced glutathione (GSH) levels were determined using the QuantiChrom™ GSH Assay Kit (BioAssay Systems) according to the instructions of the manufacturer. Briefly, control and LPS-treated U-937 cells were harvested, rinsed in cold PBS and homogenized by sonication in a solution containing 50 mM phosphate (pH = 6) and 1 mM EDTA. Lysates were centrifuged at 10,000 g for 10 min at 4°C and supernatant collected for measurement of GSH. The method comprises a colorimetric reaction of GSH with 5,5'-dithiobis(2-nitro-benzoic acid), which yields a yellow product. Optical density was measured at 415 nm in a plate reader.

### RNA isolation and real-time quantitative PCR (qPCR)

For leukocyte isolation, blood samples from MS and controls were collected in K_3_EDTA and kept not longer than 6 h at 4°C for avoiding changes in the RNA expression profile. Blood was centrifuged at 300 × g during 10 min and then, the plasma was removed. Red cells were lysed with a buffer containing 155 mM NH_4_CL, 01.mM EDTA and 10 mM KHCO_3_, pH 7.4. The blood-buffer solution was centrifuged at 300 × g during 10 min and the pellet was washed twice. The end resulting pellet of leukocytes was stored at -80°C until use.

Total RNA was isolated from U-937, rat optic nerve, and human optic nerve and leukocytes using TRIzol Reagent (Invitrogen) according to manufacturer's guidelines. Subsequently, 2 μg of RNA were retrotranscribed into cDNA using SuperScript III retrotranscriptase (200 U/μl; Invitrogen) and random hexamers as primers following manufacturer's instructions.

Gene expression levels were analyzed using qPCR. Primers were designed with PrimerExpress software (Applied Biosystems), and their sequences and gene accession numbers are provided in Additional file 2, Table S1. qPCR reactions were carried out as previously described [21] using SYBRGreen (Invitrogen) as the fluorescent reporter dye in an ABI PRISM 7000 Sequence Detection System instrument (Applied Biosystems). qPCR products were subjected to a dissociation protocol to ensure that a single product of the expected melting temperature was obtained. mRNA expression level of a given sample was calculated from a standard curve of stock cDNA obtained from the same tissue. Then, a normalization factor was calculated for each sample by GeNorm v.3.4 software based on the expression levels of 4 housekeeping genes for human samples (18s ribosomal RNA, HRPT-1, β-2 microglobulin and cyclophilin A), and 3 genes for rat samples (18s ribosomal RNA, cyclophilin A and GAPDH) [23]. Expression level of each sample was divided by this normalization factor. Data are expressed as fold change in expression compared to the mean of the matched controls. The relative abundance of the different transporters was determined by using the ΔCt method (see User Bulletin 2; Applied Biosystems).

### Western blotting, flow cytometry and immunohistochemistry

Western blot analysis of cystine/glutamate antiporter expression was done in U-937 cell cultures and in rat and mice spinal cord homogenates by conventional SDS-PAGE polyacrylamide electrophoresis as described previously [24]. The system x_c_^- ^was revealed with antibodies against the catalytic subunit xCT (0.25 μg/ml; ab37815; AbCam). Densitometric analysis was performed using the NIH Image program (*n *= 3 in triplicate).

xCT expression in human blood monocytes was analyzed by flow cytometry. PBMCs were stained using an antibody to CD14 conjugated to PE (BD Pharmigen), and an antibody to the intracellular epitope of xCT (2.1 μg/ml; ab37815; AbCam) conjugated to FITC. IgG (Sigma) was used as isotype control for xCT antibody. Extracellular staining was done in PBS buffer containing BSA, and for intracellular staining saponin was added to the previous buffer. Acquisition was performed in BSA with PBS buffer, using a Coulter EPICS Elite flow cytometer. Data were analyzed using the WinMDI v 2.9 software. Density plots were used for gating CD14^+ ^cells in PBMC populations, and then histograms for xCT-FITC were opened. The geometric mean of the fluorescence was used for statistical analysis.

xCT protein expression in rat and human spinal cord was examined by double immunofluorescence. Animals were deeply anesthetised with chloral hydrate (500 mg/kg, i.p.) and transcardially perfused with 0.1 M sodium phosphate buffer (PB), pH 7.4, followed by 4% paraformaldehyde (PFA) in the same buffer. After extracting spinal cord, tissue was postfixed 2 h in 4% PFA in PB and subsequently dehydrated in PBS containing 20% sucrose. Tissue was included in Tissue-tek resin (Sakura) in a Frigocut 2800 E cryostat (Leica) and frozen. Serial sections of 10 μm from both rat and human spinal cords were incubated with monoclonal antibodies against CD68 (1:50; DakoCytomation) for human monocyte-macrophage staining, and OX-42 (1:50; Serotec) for rat macrophage-microglia staining. Next, samples were incubated with anti-xCT antibody (5 μg/ml; ab37815; AbCam). Appropriate fluorescent secondary antibodies (Invitrogen) were used in each case. In human samples, Hoechst 33258 labeling (5 μg/ml, Sigma) was used for cell nucleus staining. Control staining was done in the absence of primary antibodies and by preincubating with peptide (ab93079; Abcam) and gave no labeling (Additional file 3, Figure S2). The same results were obtained with antibodies to xCT from Novus (NB300-317) and the staining with this antibody also disappears after preincubation with Novus peptide (NB300-317PEP) (data not shown). Images were taken by Olympus Fluoview FV500 and Leica LCS SP2 AOBs confocal microscopes.

### Statistical analysis

Data are expressed as mean ± SEM. Statistical analysis was performed using unpaired Student *t *test or ANOVA as appropriate. Correlation between xCT mRNA and EAAT2 or CD8 mRNA expression in human optic nerve was assessed by Pearson's correlation test.

## Results

### Human monocytes release glutamate through cystine/glutamate antiporter

**Figure 1:**
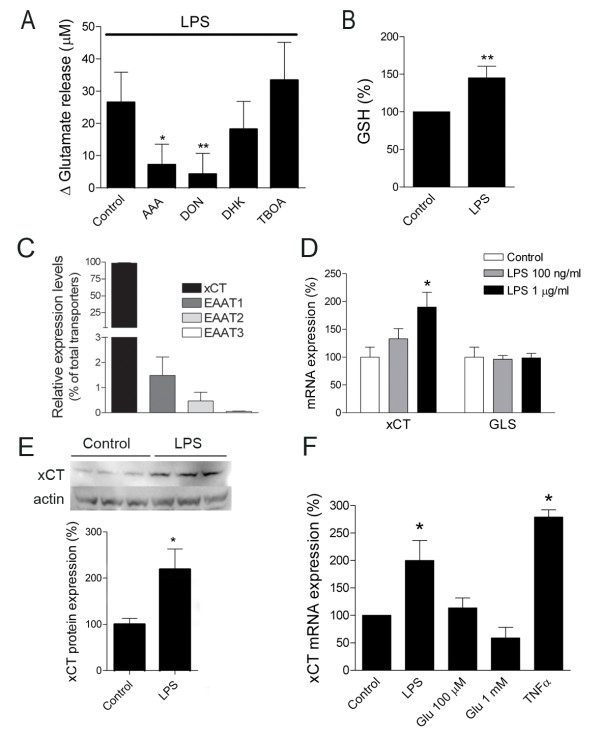
**Activated U-937 monocytes release glutamate through cystine/glutamate antiporter and show an increased expression of the xCT subunit**. A. Glutamate release by U-937 cells after activation with LPS (1 μg/ml) for 48 h in the absence and in the presence of the inhibitor of cystine/glutamate antiporter, AAA (1 mM), the glutaminase inhibitor DON (1 mM) and the inhibitors of glutamate transporters, DHK (1 mM), and TBOA (100 μM). Ordinates indicate the difference between the amount of glutamate released by LPS-activated and resting monocytes. Data are mean ± SEM from 4 independent experiments performed in triplicate. B, Intracellular glutathione levels in control U-937 cells and after activation with LPS (1 μg/ml) for 48 h. C. Relative expression of xCT antiporter and glutamate transporters EAAT1, EAAT2 and EAAT3 in CD14^+ ^monocytes. D. Histogram illustrates the increase of xCT mRNA expression, but not of glutaminase (GLS), in LPS-activated U-937 monocytes using qPCR. U-937 cells were treated with LPS (1 μg/ml) for 48 h and qPCR data were normalized using 4 housekeeping genes and GeNorm software. Data are mean ± SEM from 3 independent experiments performed in triplicate. E. Western blotting analysis shows an up-regulation of xCT protein in U-937 cells after LPS (1 μg/ml) treatment for 48 ch. Data were normalized to actin and expressed as mean ± SEM from 3 independent experiments performed in triplicate. F. xCT mRNA levels in U-937 monocyte cell line increase after LPS (1 μg/ml) treatment but not after incubation with glutamate (100 μM and 1 mM) for 48 h. Stimulation with the cytokine TNFα (10 ng/ml; 24 h) also induced a significant increase in xCT mRNA expression. qPCR data were normalized using 4 housekeeping genes and GeNorm software. Data are mean ± SEM from 3 independent experiments performed in triplicate. *, *p *< 0.05; **, *p *< 0.01.

We have previously demonstrated that activation of U-937 monocytes induces glutamate release [13]. So, we now analyzed the mechanism of glutamate release in U-937 cells as well as in peripheral blood monocytes *in vitro*. LPS-activated U-937 monocytes (LPS 1 μg/ml, 48 h) showed a significant increase in glutamate levels (*n *= 4; *p *< 0.001; Figure 1A), as previously described [13]. Incubating cells with the non competitive blocker of glutamate transporters DL-threo-beta-benzyloxyaspartate (TBOA; 100 μM) or with dihydrokainate (DHK; 1 mM), a selective inhibitor of EAAT2 glutamate transporter, did not block LPS-induced glutamate release by monocytes (*n *= 4; Figure 1A), excluding any role of glutamate transporters in such glutamate release. In contrast, incubating cells with aminoadipic acid (AAA, 1 mM), an inhibitor of cystine/glutamate antiporter [25,26], significantly reduced glutamate release by LPS-activated monocytes (*n *= 4; *p *= 0.02; Figure 1A), indicating that the x_c_^- ^system is the main glutamate release mechanism in monocytes. Accordingly, intracellular glutathione levels were increased after LPS treatment (n = 3; p = 0.03; Figure 1B), as a result of an increased function of xCT antiporter.

Finally, 6-diazo-5-oxo-L-norleucine (DON; 1 mM), an inhibitor of glutaminase enzyme, also inhibited significantly glutamate release by monocytes (*n *= 4; *p *= 0.02; Figure 1A). Glutaminase is the key enzyme to replenish glutamate pool in neuronal presynaptic terminals. Similarly, it is possible that blocking glutaminase in monocytes depletes the pool of glutamate that is released after their activation with LPS.

### Activation of human monocytes with LPS upregulates cystine/glutamate antiporter expression

Because the system x_c_^- ^is modulated by oxidative stress [27], we next examined whether LPS modulated system x_c_^- ^expression in monocytes. System x_c_^- ^is a heteromeric protein complex consisting on a catalytic L chain (xCT) and a regulatory H chain (4F2hc) [27]. In control patients, the levels of xCT in CD14^+ ^monocytes are much higher than the levels of the major glutamate transporters EAAT1 and EAAT2 (Figure 1C). We then analyzed the expression of xCT in U-937 cells after their activation with LPS (1 μg/ml, 48 h) by quantitative PCR (qPCR) and Western blot. LPS activation (1 μg/ml) induced an increase in xCT mRNA expression (*n *= 5; *p *= 0.014; Figure 1D). Accordingly, Western blot analysis revealed a two-fold increase in xCT protein levels (*n *= 3; *p *= 0.01; Figure 1E). Upregulation of xCT expression is not secondary to glutamate homeostasis alteration since the incubation with glutamate (100 μM to 1 mM; 48 h) did not change xCT mRNA expression (*n = 3*; Figure 1F), suggesting that extracellular glutamate increase is correlative, not causative, of xCT upregulation. In addition, the expression of other enzymes involved in glutamate metabolism, such as glutamine synthetase, was not change after LPS treatment (Figure 1D). Similar to LPS, activation with TNFα also induced an increase in xCT mRNA expression (Figure 1F). Altogether, these results show that LPS activation enhances xCT expression and function in U-937 monocyte cell line.

**Figure 2:**
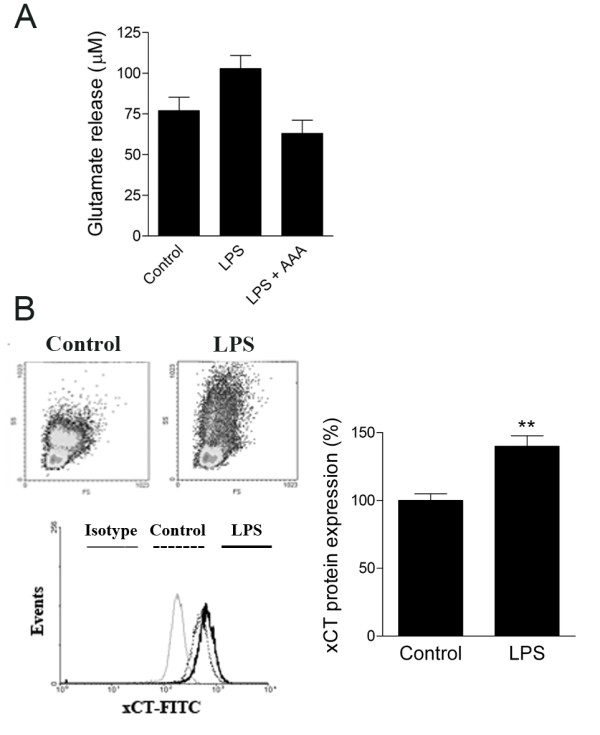
**Activation of human peripheral blood monocytes by LPS induces glutamate release through the cystine/glutamate antiporter and xCT upregulation**. A. Monocytes, isolated by MACS, showed an increase in glutamate release after their activation with LPS (1 μg/ml, 48 h). This effect is prevented in the presence of AAA (1 mM), an inhibitor of the cystine/glutamate antiporter. B. Flow cytometry analysis of peripheral blood monocytes (CD14-PE) reveals a change in the morphology of monocyte population after their activation with LPS (left-up). Staining with xCT-FITC demonstrates higher expression of this exchanger in LPS-activated CD14^+ ^monocytes (left-down and right). *, *p *< 0.05; **, *p *< 0.01; #, *p *< 0.05 vs. LPS treatment.

We then examined if the aforementioned features are also present in blood monocytes freshly isolated by MACS. After isolation, cells were maintained in culture for 96 h and subsequently activated with LPS (1 μg/ml, 48 h). Similarly to U-937 cell line, monocytes showed an increase of 33.5% in glutamate levels after their activation with LPS (*p *= 0.04; Figure 2A), which was blocked in the presence of AAA (n = 3; *p *= 0.01; Figure 2A). To confirm changes in xCT expression in LPS-activated human monocytes we carried out flow cytometry analysis. LPS-treated monocytes, not lymphocytes, showed a change in the granularity, a parameter indicative of the activated state of monocytes (Figure 2B left-up). The analysis of xCT expression was performed by gating CD14^+ ^monocytes. Staining cells with xCT antibodies showed a clear shift versus the IgG isotype control, indicating that the staining with this antibody was specific (Figure 2B left-bottom). Similar to the results obtained in the U-937 cell line, there was an increase in xCT expression in CD14^+ ^cells after their activation with LPS (*n *= 5; *p *= 0.0098; Figure 2B right). Thus, LPS activation also enhances xCT expression and function in peripheral blood monocytes.

### xCT expression is increased in EAE

Since glutamate levels are reported to be altered in MS disease [12,13,28] and monocyte infiltration and microglial activation are MS hallmarks [29], we hypothesized that the expression of system x_c_^- ^could be altered in MS disease. To test this hypothesis, we analyzed the expression of xCT in the spinal cord of rats after induction of acute EAE, an experimental model of MS which reproduces the inflammatory and immune component of the disease.

**Figure 3:**
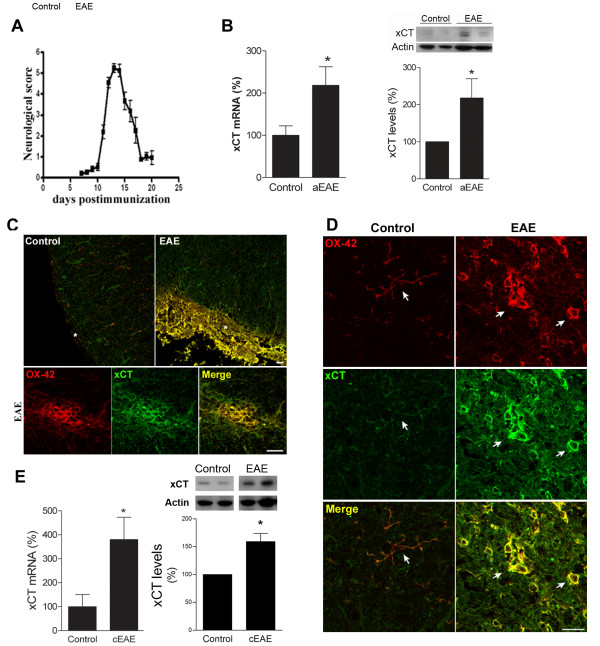
**xCT expression is increased in the CNS of rats with EAE**. A. Histogram showing the neurological score during the course of acute EAE induced in Lewis rats by immunization with myelin basic protein. The peak of neurological disability was at day 14 post-immunization, which was selected for obtaining tissue samples. B. xCT mRNA (left) and protein (right) expression in spinal cord from control and acute EAE rats, as assessed by qPCR and Western blot analysis. Data are referred to mean expression level of controls (*n *= 5-6). C. Double immunofluorescence for xCT (green) and OX-42 (red), a marker of microglia and infiltrating macrophages. OX42^+ ^cells express high xCT levels in acute EAE. Both meninges (asterisk in top) and infiltrating cells (bottom) in inflammatory foci show high levels of xCT in rat spinal cord with EAE as compared to controls. D. Microglial cells (OX42^+ ^cells) of EAE rats have higher xCT levels in spinal cord than controls. Notice the difference between resting microglia in control rats, with ramified morphology (arrows in control) and microglia in EAE showing round shaped morphology, characteristic of its activated state (arrows in EAE). Scale bar = 20 μm.E. xCT mRNA (left) and protein (right) expression in spinal cord from control and chronic EAE mice, assessed by qPCR and Western blot analysis. Data are referred to mean expression level of controls (*n *= 5).

Rats immunized with myelin basic protein showed maximal motor deficits at around 14 days postimmunization (Figure 3A). At that stage, expression of mRNA encoding xCT was more abundant in spinal cord samples from rats with EAE than in control animals (*n *= 6; *p *= 0.013; Figure 3B left). Accordingly, xCT protein levels were significantly increased in EAE rats (n = 5; p < 0.05; Figure 3B right). We also analysed the expression of xCT in the spinal cord by double immunofluorescence using antibodies to OX-42, a marker of microglia lineage. No immunolabeling was detected after preincubation xCT antibodies with the corresponding peptide (Additional file 3, Figure S2), demonstrating the specificity of the immunofluorescence signal. xCT expression was dramatically increased in the spinal cord of rats with EAE. The expression was particularly increased in meninges in EAE animals, showing clear signs of inflammation (asterisk in Figure 3C top right). There was also a clear increase of xCT in OX42^+ ^infiltrating cells, which were organized in clusters around vessels (Figure 3C bottom). In addition, activated microglial cells in EAE rats showed a massive upregulation of xCT (Figure 3D). Activated microglia was identified by their characteristic ameboid shape in contrast with the ramified appearance of resting microglia in control animals (arrows in Figure 3D). Increases in xCT expression at the level of RNA and protein were also detected in the chronic EAE mice (n = 5 for RNA and protein; p < 0.05; Figure 3E), a model that reproduces the chronic and progressive phase of MS.

### xCT expression is increased in MS

**Figure 4:**
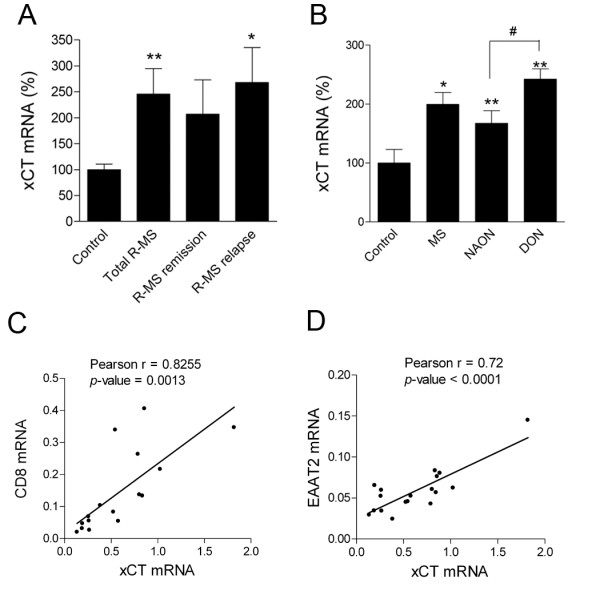
**xCT mRNA expression is increased in MS patients**. A. Data show a significant increase in xCT mRNA expression in leukocytes in relapsing-MS, which is more prominent during relapses. Controls, *n *= 39; Total R-MS, *n *= 42; R-MS in remission, *n *= 24; R-MS in relapse, *n *= 18. B. Data show a significant increase in xCT mRNA in human optic nerve from MS patients as compared to matched controls. Damage optic nerves (DON), showing macroscopic plaques, atrophy and/or optic neuritis have a significantly higher increase in xCT mRNA. *, *p *< 0.05; **, *p *< 0.01; #, *p *< 0.05. C, D. xCT mRNA expression correlates with CD8 and EAAT2 mRNA expression in MS optic nerve. Pearson *r *= 0.83; n = 10; *p *= 0.0013 for CD8 vs. xCT. Pearson *r *= 0.81; *n *= 10; *p *= 0.005 for EAAT2 vs. xCT.

We next checked whether system x_c_^- ^expression is similarly altered in MS disease. We analyzed the expression of xCT in peripheral blood and in postmortem optic nerve samples from MS patients by quantitative PCR. xCT mRNA levels showed a 2.5 average fold increase in isolated leukocytes from MS patients (*n *= 52) compared to controls (*n *= 25; *p *= 0.003; Figure 4A). In turn, the increase in xCT levels was higher in R-MS patients having a clinical relapse (*n *= 24; *p *= 0.016; Figure 4A) than during remission (*n *= 28; *p *= 0.055; Figure 4A). Elevated xCT expression was also observed in CNS samples. Thus, the levels of xCT mRNA in optic nerve (ON) from MS patients showed a two fold increase compared to matched controls (*n *= 16; *p *= 0.027; Figure 4B). The increase in xCT mRNA expression was significantly higher in damaged optic nerves showing macroscopic plaques, atrophy and/or optic neuritis. Moreover, levels of xCT mRNA showed a high correlation with CD8 mRNA (Pearson *r *= 0.83; n = 16; p = 0.0013, Figure 1C), a marker of infiltration and lymphocyte activity, suggesting that lesion activity correlates with xCT expression. Interestingly, the levels of xCT expression in the ON of MS patients correlated positively with the levels of EAAT2 reported previously [21] (Pearson *r *= 0.81; *n *= 10; *p *= 0.005; Figure 1D). Because an enhanced xCT expression is associated to an increase in extracellular glutamate in monocytes *in vitro*, the increase in glutamate transporter expression could be an adaptive response to counteract the excess of glutamate driven by xCT in MS.

**Figure 5:**
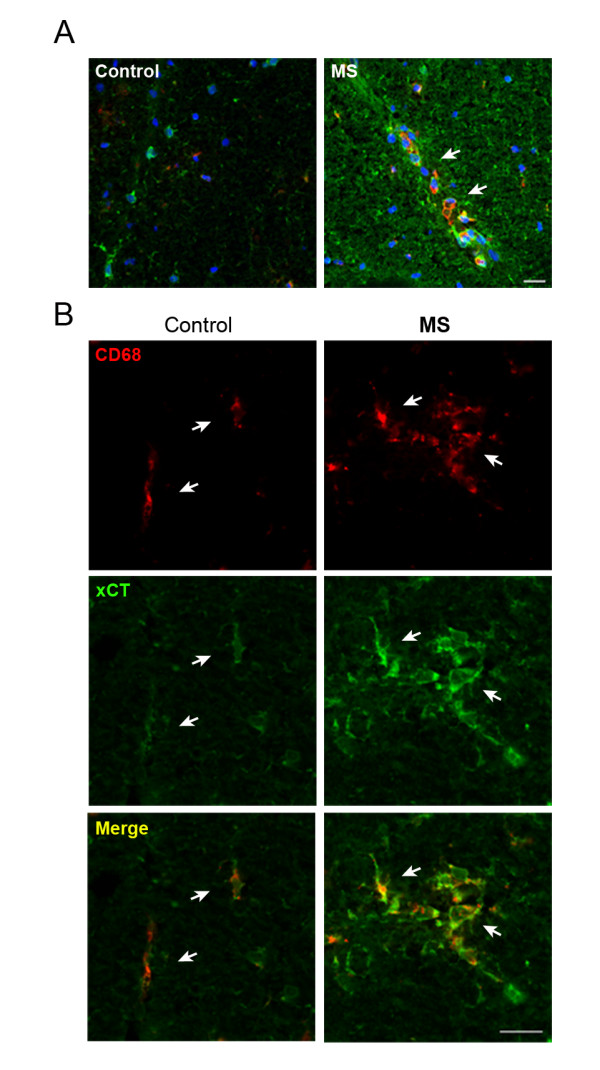
**xCT expression is enhanced in CD68^+ ^cells from MS spinal cord**. A. Triple immunofluorescence staining for xCT (green), CD68 (red) and Hoechst 33258 (blue) in spinal cord of control (left) and MS patients (right). A high expression of xCT was detected in CD68^+ ^infiltrating macrophages (arrows) associated with blood vessels, which are virtually absent in controls. Note that overall xCT expression is enhanced in MS tissue. B. CD68^+ ^cells (arrows) show enhanced xCT expression in MS patients as compared to controls. CD68^+ ^macrophages are round shaped and form clusters in MS patients, whereas in controls, CD68^+ ^cells appear isolated and long shaped. Scale bar = 50 μm.

Finally, we performed double immunofluorescence analysis of xCT expression in the spinal cord of MS patients and controls. Overall, we observed a higher expression of xCT in the CNS of MS patients than in controls. In particular, infiltrating CD68^+ ^macrophages revealed higher xCT expression in MS patients than in controls (Figure 5). Thus, CD68^+ ^cells located in blood vessels (Figure 5A) or nearby, and usually forming clusters, showed a clear xCT overexpression in MS patients (Figure 5B). These results are consistent with the increase of xCT expression in EAE animals, and suggest that altered xCT expression may have a pathophysiological role in MS disease.

## Discussion

In the current study we have shown that blood monocytes release glutamate through the x_c_^- ^system and that its constitutive expression and function is upregulated by activation with LPS. In addition, we provide evidence that xCT expression is increased in activated macrophages-microglia in the CNS, both in EAE and in MS, as well as in leukocytes from MS patients.

The findings described here point to system x_c_^- ^as a major glutamate release mechanism in activated monocytes. This is consistent with previous observations illustrating the expression of the x_c_^- ^system in microglia [22] and macrophages [25,30]. Importantly, we show that the increase in glutamate release after the inflammatory stimulus is due to an elevation in the expression of xCT, the catalytic subunit of the system x_c_^-^, which is constitutively expressed in human monocytes. These results are in keeping with other studies reporting that mouse macrophages induce x_c_^- ^expression in response to both LPS and oxygen [31-33], a mechanism that allows replenishment of the glutathione pool during inflammatory conditions and oxidative stress. Glutamate did not change xCT expression by itself, excluding the possibility that xCT expression changes could be adaptive changes to extracellular glutamate accumulation. Altogether, the expression of the x_c_^- ^system in monocyte-macrophage-microglia lineage provides a direct link between glutamate homeostasis alteration and inflammation.

We also provide evidence that, like in neurons, glutaminase is a key enzyme to replenish the glutamate pool necessary to efficiently release glutamate in monocytes. In accordance, other studies have reported that inhibition of glutaminase prevents from glutamate release in activated microglia [34,35]. Interestingly, glutaminase expression is increased in macrophages and microglia that are close to dystrophic axons in active MS lesions [36], suggesting a possible role of this enzyme in glutamate homeostasis alteration and MS pathophysiology. Indeed, the implication of enhanced glutaminase expression and activity in MS is sustained by the fact that its inhibition attenuates EAE clinical symptoms, possibly by diminishing oligodendroglial cell death and axonal damage since inflammatory cell infiltration is not reduced [37].

We observed an increased xCT expression in OX-42^+ ^cells (microglia, meningeal cells and dendritic cells) in the spinal cord of rats with acute EAE. Meninges constitutively express xCT, which contributes to the maintenance of the redox state by recycling cystine to cysteine in the CSF and plasma [38]. In addition, cellular infiltrates, which are typically composed by infiltrating macrophages and dendritic cells [17], and usually enter the CNS through blood vessels [39], also showed higher xCT level in EAE. Finally, resident microglia, distinguished by OX-42^+ ^staining and by their characteristic rounded morphology indicative of activated state, showed a clear increase of xCT during EAE. Altogether, xCT overexpression in OX-42^+ ^cells during EAE, may serve to counteract oxidative stress during inflammatory conditions. However, an increased uptake of cystine leads to an enhanced glutamate release, which could have negative consequences to oligodendroglial survival. Indeed, an enhanced activity of x_c_^- ^system secondary to microglial activation has been reported to induce oligodendrocyte cell death by glutamate receptor overstimulation [22].

Consistent with the findings in the EAE model, MS patients show an increased xCT expression in spinal cord macrophages which are located in blood vessels and form cell clusters. Moreover, higher xCT expression in leukocytes during the course of MS relapses suggests dynamic changes in response to inflammatory activity. An increased expression of xCT or an altered function of x_c_^- ^system has been described in other CNS disorders [40]. In malignant brain tumors, glutamate release by glioma cells through the x_c_^- ^system contributes to glioma-induced peritumoral cell death, and inhibition of its activity lessens neurodegeneration and alleviates perifocal edema [41]. In addition, an increased expression of xCT has been described in an animal model of Parkinson's disease [42] and in reactive microglia located in amyloid plaques of transgenic mice expressing mutant human amyloid precursor protein, as well as in wild-type mice injected with amyloid-β [26]. Altogether data indicate that x_c_^- ^system expression is altered as a consequence of inflammation and oxidative stress in CNS disorders with an important inflammatory component. Because system x_c_^- ^plays an essential role in the antioxidant defense of the cell [38,43], more detailed studies are needed to determine whether the system constitutes a new therapeutic target for these disorders.

## Conclusions

This study provides the first evidence that activated monocytes/macrophages/microglia release glutamate through cystine/glutamate antiporter by increasing xCT expression, as assessed by *in vitro *assays as well as in both EAE and MS. These findings support the idea that system x_c_^- ^acts as a link between inflammation and glutamate excitotoxicity, and that xCT is a potential target to attenuate glutamate excitotoxicity in neurodegenerative diseases undergoing inflammation.

## Abbreviations

MS: Multiple sclerosis; iGluRs: ionotropic glutamate receptors; EAE: experimental autoimmune encephalitis; x_c_^-^: cystine/glutamate antiporter; EAAT: excitatory amino acid transporter; R-MS: relapsing MS; RR-MS: relapsing-remitting MS; SP-MS: secondary-progressive MS; NAON: normal appearing optic nerve; DON: damaged optic nerve; qPCR: real-time quantitative PCR.

## Competing interests

The authors declare that they have no competing interests.

## Authors' contributions

OP carried out all the experiments and contributed to the manuscript. FN performed the Western blot analysis in EAE and the GSH measurement experiments. MD designed the study, coordinated the experiments, prepared the figures, and contributed to the manuscript. PV and AR-A collected samples from MS patients, analyzed data, and discussed the manuscript. CM designed the study, coordinated the experiments and contributed to the manuscript. All authors have read and approved the final version of the manuscript.
